# Œil et Maladie de Behçet: profil épidémiologique, clinique, thérapeutique et évolutif

**DOI:** 10.11604/pamj.2019.33.116.17111

**Published:** 2019-06-14

**Authors:** Amine Razzak, Anass Kassimi, Adil Mchachi, Leila Benhmidoune, Abderrahim Chakib, Rayad Rachid, Mohamed Elbelhadji, Abdelouahed Amraoui, Hassan Elkabli

**Affiliations:** 1 Service d'Ophtalmologie Adultes, Hôpital 20 Août 1953, Centre Hospitalier et Universitaire Ibn Rochd, Casablanca, Maroc; 2 Service de Médecine Interne, Hôpital Ibn Rochd, Centre Hospitalier et Universitaire Ibn Rochd, Casablanca, Maroc

**Keywords:** Epidémiologie, uvéites, vascularites, maladie de Behçet, Epidemiology, uveitis, vasculitis, Behcet’s disease

## Abstract

Le but de ce travail est d'étudier le profil épidémiologique, clinique, thérapeutique et évolutif des manifestations oculaires au cours de la Maladie de Behçet. C'est une étude rétrospective descriptive étalée sur une période d'un an et demi entre janvier 2015 et juin 2016 analysant les dossiers de 121 patients suivis à la consultation spécialisée de la maladie de Behçet. La moyenne d'âge était de 35 ans, 63,6% des patients étaient de sexe masculin, l'atteinte oculaire était inaugurale dans 24% des cas. Les patients présentaient une uvéite antérieure (7,4%), une uvéite postérieure (15,7%), une vascularite (19%), des synéchies irido-cristalliniennes (17,5%), un œdème maculaire (7,4%), une atrophie optique (4,1%), un œdème papillaire (2,5%) et une ischémie rétinienne périphérique (1,7%). Dans notre série 41,3% des patients étaient sous colchicine, 23,1% sous corticoïdes par voie orale, 9% sous corticoïdes par voie intraveineuse, 4,9% sous corticoïdes topiques, 8,2% sous immunosuppresseurs et 5,8% sous anti-vitamine K. Sur un recul moyen d'une année, 40% des patients avaient une acuité visuelle stable sous traitement, 23% ont présenté une baisse significative de l'acuité visuelle dont 5% des cas de cécité absolue. Une prise en charge thérapeutique correcte permet de juguler rapidement la poussée inflammatoire et de réduire la fréquence et la gravité des récidives ce qui se traduit par une amélioration du pronostic visuel de nos patients par rapport à des séries antérieures.

## Abstract (en)

This study aims to analyze the epidemiological, clinical, therapeutic and evolutionary profile of ocular manifestations in patients with Behçet’s disease. We conducted a retrospective, descriptive study of the medical records of 121 patients managed by specialists with expertise in this disease over a period of one year and a half between January 2015 and June 2016. The average age of patients was 35 years, 63.6% of patients were male, ocular involvement was inaugural in 24% of cases. Patients had anterior uveitis (7.4%), posterior uveitis (15.7%), vasculitis (19%), irido-crystalline synechias (17.5%), macular edema (7.4%), optic atrophy (4.1%), papillary edema (2.5%) and peripheral retinal ischemia (1.7%). In our series, 41.3% of patients were under colchicine, 23.1% under oral corticosteroids, 9% under intravenous corticosteroids, 4.9% under topical corticosteroids, 8.2% under immunosuppressive drugs and 5.8% under vitamin K antagonists. After an average follow-up of 1 year, 40% of patients had stable visual acuity while receiving treatment, 23% had a significant decrease in visual acuity and 5% of cases had complete vision loss. Adequate therapy enables quick containment of the infection and decreases the frequency and severity of recurrences, thus leading to an improvement of the visual prognosis in our patients compared with outcomes in some previous case serie.

## Introduction

La maladie de Behçet (MB) est une affection inflammatoire systémique d'étiologie inconnue évoluant par poussées. La manifestation oculaire la plus fréquente est une panuvéite associée à une vascularite, il s'agit d'une atteinte grave pouvant mettre en jeu le pronostic visuel [1]. L'atteinte oculaire au cours de cette maladie est fréquente au Maghreb, cependant, peu de publications marocaines se sont penchées sur ce sujet [2]. Le but de cette étude est d'établir le profil épidémiologique, clinique, thérapeutique et évolutif des manifestations oculaires au cours de la maladie de Behçet dans notre région.

## Méthodes

Il s'agit d'une étude rétrospective menée entre janvier 2015 et juin 2016 dans notre service d'ophtalmologie, colligeant tous les patients adultes suivis dans notre consultation spécialisée de la maladie de Behçet. Tous les patients ont bénéficié d'un interrogatoire détaillé et d'un examen ophtalmologique complet, comportant une mesure de la meilleure acuité visuelle corrigée, une mesure du tonus oculaire, un examen à la lampe à fente et un examen du fond d'œil au verre à 3 miroirs de Goldmann. Les examens complémentaires (angiographie rétinienne à la fluorescéine, tomographie en cohérence optique (OCT), échographie oculaire) ont été demandés chaque fois que leurs indications se posent. Tous les patients ont bénéficié d'une consultation en médecine interne, d'autres examens spécialisés ont été demandés si besoin en collaboration avec les internistes. Le diagnostic de la MB a été établi selon les critères de l'International Study Group for Behcet's Disease (ISG) [3].

## Résultats

Le nombre total de patients était de 121 (198 yeux) dont 63,6% étaient des hommes, la moyenne d'âge était de 35 ans avec un écart-type de 12,8. Le délai moyen entre le diagnostic de la maladie de Behçet et l'apparition de la 1ère atteinte oculaire était de 5 ans. Dans notre série, 33% des patients présentaient au moins 2 épisodes d'œil rouge à répétition par an, l'atteinte oculaire était inaugurale dans 24% des cas. Sur le plan clinique, l'acuité visuelle était supérieure à 5/10 dans 8,5% des cas, entre 1/10 et 5/10 dans 62,1% des cas, inférieure à 1/10 dans 26,4% des cas, une absence de perception lumineuse dans 4,1% des yeux. Concernant les manifestations cliniques de la maladie de Behçet qui sont résumées au tableau 1, la forme anatomique la plus fréquente de l'atteinte oculaire était la panuvéite (20%) tandis que les complications étaient dominées par la cataracte et les synéchies irido-cristaliniennes (Tableau 1). Les manifestations extraoculaires de la maladie de Behçet sont résumées dans le tableau 2, elles étaient dominées par l'aphtose buccale (critère obligatoire) et l'aphtose génitale chez 41,3% des patients (Tableau 2). Dans notre série, 50% des patients étaient sous colchicine, 28% sous corticoïdes par voie orale, 11% sous corticoïdes par voie intraveineuse, 10% sous immunosuppresseurs et 5,8% sous antivitamine K. Sur un recul moyen d'une année, 40% des patients ont gardé une acuité visuelle stable sous traitement, 23% ont présenté une baisse significative de l'acuité visuelle dont 5% des cas de cécité absolue. Seul 6,6% ont présenté une amélioration de l'acuité visuelle sous traitement et 30% des patients ont été perdus de vue.

**Tableau 1:** Manifestations ophtalmologiques et complications de la maladie de Behçet

	Nombre d’yeux (n=198)	Pourcentage %
Panuvéite	40	20
Uvéite antérieure	14	7
Uvéite intermédiaire	6	3
Uvéite postérieure	30	15,1
Signes de vascularite	37	18,7
Œdème maculaire	15	7,5
Œdème papillaire	5	2,5
Atrophie optique	18	9
Synéchies irido-cristaliniennes	34	17,2
Cataracte	60	30,3
Hypertonie oculaire	11	5,6
Ischémie rétinienne	3	1,5
Décollement de rétine	18	9,1

**Tableau 2:** manifestations extraoculaires de la maladie de Behçet

	Nombre de patients (n=121)	Pourcentage %
Aphtose buccale	121	100
Aphtose génitale	50	41,3
Lésions cutanées	25	20,6
Atteinte ostéo articulaire	29	24
Atteinte Neurologique	11	9,1
Atteinte gastro-intestinale	9	7 ,4
Atteinte vasculaire	16	13,2

## Discussion

La maladie de Behçet a été décrite pour la première fois par Hulusi Behçet en 1937 qui a rapporté une triade diagnostique composée de l'ulcération buccale récidivante, de l'ulcération génitale et de l'uvéite [4]. Plusieurs critères diagnostiques de la maladie ont été proposés, jusqu'à 1990 lorsque l'International Study Group for Behçet Disease établit des critères diagnostiques cliniques [3]. Ainsi, le diagnostic de maladie de Behçet, selon cette classification, nécessite la présence d'une aphtose buccale récidivante, associée à au moins deux critères parmi les quatre critères suivants: l'aphtose génitale, l'uvéite, la pseudo-folliculite, et le « pathergy test ». Cette maladie touche les deux sexes, avec une plus grande fréquence et gravité chez l'homme. L'âge de début de la maladie se situe le plus souvent entre 25 et 30 ans [5]. Dans notre série 63,3% des patients étaient de sexe masculin, avec une moyenne d'âge de 35 ans. L'atteinte oculaire au cours de la maladie survient dans 67 à 95% des cas; elle est parfois décalée dans le temps de 1 mois à 2 ans. En général, les premières poussées inflammatoires sont plutôt unilatérales et antérieures. Secondairement, les récidives intéressent le segment postérieur et deviennent bilatérales. L'uvéite peut être inaugurale dans 10 à 20% des cas, parfois, elle apparaît deux à 3 ans après l'aphtose buccale [6]. L'atteinte oculaire était inaugurale chez 24% de nos patients, ce qui est concordant avec les données de la littérature. Bien qu'étant rarement la première manifestation, l'uvéite est fréquemment l'atteinte conduisant au diagnostic de la maladie, elle se présente sous forme d'une panuvéite non granulomateuse bilatérale, associée à une vascularite rétinienne. Une minorité de patients, particulièrement des femmes, se présentent avec une uvéite antérieure isolée [1,7,8].

Tugal-Tutkun a rapporté dans une étude rétrospective incluant 880 patients, un taux de panuvéite de 60,2% des patients, l'uvéite postérieure était la seconde forme la plus fréquente (51,6%) [9]. Ces données sont compatibles avec celles obtenues dans notre étude, en effet, 20% de nos patients présentaient une panuvéite, tandis que seul 7% présentaient une uvéite antérieure isolée. Dans la littérature, l'œdème maculaire diffus cystoïde ou non cystoïde est la complication la plus fréquente de la maladie, il conditionne le pronostic visuel à long terme et peut aboutir à la formation d'un trou maculaire [10]. La cataracte constitue la complication la plus fréquente dans notre étude, elle est secondaire à l'uvéite ou au traitement corticoïde topique ou général, l'aspect le plus fréquent est celui de cataracte sous capsulaire postérieure [11]. Les autres complications peuvent concerner le segment antérieur: synéchies postérieures, hypertonie oculaire, glaucome secondaire; ou le segment postérieur: occlusions de branches veineuses rétiniennes, occlusions de la veine centrale de la rétine, occlusion de branche artérielle rétinienne, néovascularisation prérétinienne ou prépapillaire associée ou non à une ischémie rétinienne périphérique [12]. D'autres complications moins fréquentes ont été rapportées: hémorragie intravitréenne, déhiscences rétiniennes, décollement de rétine et phtyse du globe [1] (Tableau 3) [9, 13, 14]. La maladie de Behçet est caractérisée par une vascularite occlusive pouvant intéresser plusieurs organes. L'aphtose bipolaire (buccale et génitale), l'atteinte oculaire et les manifestations cutanées et ostéoarticulaires sont de loin les plus fréquentes [1]. Selon les critères de l'International Study Group for Behçet's Disease (ISBD), le diagnostic de maladie de Behçet nécessite la présence d'une aphtose buccale récidivante avec plus de 3 épisodes par an, en plus de deux critères parmi quatre, qui sont: l'aphtose génitale, l'uvéite, la pseudo-folliculite, et le « pathergy test ». Dans la série de Tugal-Tutkun, tous les patients présentaient une aphtose buccale récidivante, une aphtose génitale dans 59,8% des cas, une arthrite dans 34% des cas, une psoeudofolliculite (31,6%), un érythème noueux (23,6%). Dans notre étude, en plus de l'aphtose buccale qui était un critère obligatoire, 41,3% des patients présentaient une aphtose génitale, 24% des manifestations ostéoarticulaires, et 20,6% de lésions dermatologiques.

**Tableau 3:** les complications oculaires de la maladie de Behçet

Série	Cataracte (%)	Atrophie optique (%)	Synéchies irido-cristalliniennes (%)	Décollement de rétine (%)
Tugal-Tutkun *et al.* [9] Turkey 2004 (N=880)	38,5	23,6	26,1	1,4
Khairallah *et al.* [13] Tunisia 2008 (N = 62)	31,5	13.5	32,4	0,9
Yang *et al.* [14] China 2008 (N = 437)	77,4	16,2	23,9	10,7
Notre Série	30,6	9	17,5	9,1

Bien que le traitement de la MB reste à ce jour très empirique, il a été bien démontré qu'un traitement précoce et efficace des poussées aiguës inflammatoires et la prévention des rechutes améliorent nettement le devenir de la maladie [15]. Il est largement admis qu'une inflammation intraoculaire nécessite une corticothérapie prolongée associée à un immunosuppresseur afin d'éviter les récidives qui peuvent mettre en jeu le pronostic visuel. En cas d'uvéite antérieure, la corticothérapie sera administrée par voie locale en collyre, selon un rythme d'administration proportionnel à la sévérité de l'inflammation du segment antérieur [6]. L'atteinte du segment postérieur requiert l'utilisation de corticoïdes et immunosuppresseurs, en premier lieu, l'azathioprine [16]. La corticothérapie générale par voie orale est prescrite, à la dose de 1 mg/kg par jour, elle peut être précédée par des bolus de méthylprednisolone à la dose de 0,5 à 1 g/j pendant 3 jours. L'azathioprine utilisée à la dose de 2 à 2,5 mg/kg par jour est un bon traitement d'épargne cortisonique qui semble intéressant dans les atteintes oculaires postérieures peu sévères. Selon les recommandations de l'EULAR, tout patient atteint de maladie de Behçet avec atteinte du segment postérieur doit bénéficier d'un traitement associant l'azathioprine et les corticoïdes. En cas d'atteinte oculaire sévère et/ou une atteinte rétinienne (vascularite rétinienne ou atteinte maculaire), le traitement se fait soit par la ciclosporine A, soit par l'infliximab en association à l'azathioprine et aux corticoïdes. L'association IFN-alpha avec ou sans corticoïdes est aussi une alternative thérapeutique possible [17]. Les anti-TNF alpha représentent une alternative thérapeutique séduisante dans les uvéites sévères et réfractaires aux immunosuppresseurs, ils sont rapidement, efficaces mais ils nécessitent une prescription prolongée avec un coût élevé, ce qui a limité son utilisation dans notre série. Classiquement, la cécité survient dans environ 50% des cas dans les 5 ans suivant le premier signe oculaire, cependant, grâce aux avancées thérapeutiques dans la prise en charge de l'inflammation intraoculaire, le pronostic visuel s'est amélioré dans les séries de patients présentés après les années 1990 [9]. Dans notre série, la baisse de l'acuité visuelle a été rapporté chez 23% des patients sur un recul d'un an.

## Conclusion

L'atteinte oculaire constitue, non seulement un critère pour le diagnostic de la maladie de Behçet, mais aussi un élément de gravité car elle conditionne le pronostic. Le pronostic visuel au cours de la maladie de Behçet dépend de la précocité prise en charge des patients qui peut être influencée, en fonction des pays, par des considérations socio-économiques et culturelles. Une prise en charge thérapeutique correcte permet de juguler rapidement la poussée inflammatoire et de réduire la fréquence et la gravité des récidives ce qui se traduit par une amélioration du pronostic visuel de nos patients par rapport à des séries antérieures. Néanmoins, la Maladie de Behçet reste toujours une affection grave à fort potentiel cécitant malgré tous les progrès réalisés au cours des dernières années.

### État des connaissances actuelles sur le sujet

- La maladie de Behçet est fréquente dans les pays du bassin méditerranéen et en Asie;
- La forme clinique la plus fréquente est une panuvéite non granulomateuse bilatérale symétrique ou non.

### Contribution de notre étude à la connaissance

- Très peu de publications ont étudié le profil de la maladie de Behçet au Maroc;
- La cataracte constitue la complication la plus fréquente de la maladie dans notre contexte.

## Conflits des intérêts

Les auteurs ne déclarent aucun conflit d'intérêts.
